# Interfacial Characteristics and Mechanical Properties of TiAl4822/Ti6Al4V Metal–Intermetallic Laminate Composite Prepared Through Vacuum Hot Pressing

**DOI:** 10.3390/ma18040898

**Published:** 2025-02-19

**Authors:** Jianwen Qin, Shouyin Zhang, Zhijian Ma, Baiping Lu

**Affiliations:** School of Materials Science and Engineering, Nanchang Hangkong University, Nanchang 330063, China; 13534842524@163.com (J.Q.); mazhijian2020@163.com (Z.M.); 30012@nchu.edu.cn (B.L.)

**Keywords:** TiAl4822/Ti6Al4V, metal–intermetallic laminate composites, vacuum hot pressing

## Abstract

In this work, the TiAl4822/Ti6Al4V metal–intermetallic laminate (MIL) composite was fabricated using vacuum hot pressing (VHP). The interfacial morphologies and mechanical properties of the composites were investigated. No discernible defect was observed in the well-bonded interface region. This interface region comprised two distinct areas: the Ti_2_Al (6 μm) region near the TiAl layer and the Ti_3_Al (4 μm) region near the Ti6Al4V layer. Electron backscatter diffraction analysis revealed that dynamic recrystallization (DRX) took place at the interface during the hot pressing process. The ductile brittle nature of Ti6Al4V and TiAl4822 layers and the formation of fine grains within the interface are conducive to enhancing toughness and tensile strength. Room temperature tensile testing exhibited that the tensile strength of TiAl4822/Ti6Al4V MIL composite was 636.9 MPa, approximately 225 MPa higher than single TiAl4822 alloy. The Ti6Al4V layer, as well as the formation of fine grain interface, effectively inhibited further propagation of the main crack through crack passivation, crack deflection, and load transformation. The bending strength of the TiAl4822/Ti6Al4V MIL composite was 1114.1 MPa. The fracture toughness of the TiAl4822/Ti6Al4V MIL composite reached 33.15 MPam^1/2^, which increased by 78.2% compared with single TiAl4822 alloy.

## 1. Introduction

With the rapid development of the aerospace field, high-temperature materials are required to possess high strength, high rigidity, lightness and high-temperature resistance [1,2,3]. The substitution of conventionally used heavy Ni-based superalloys with lighter alloys is considered a viable alternative to enhance efficiency. In this context, intermetallic compounds, particularly TiAl-based alloys, represent a promising approach for achieving improved performance and reduced weight [4]. TiAl alloys, as a novel class of structural materials, are ideal candidate materials for aerospace applications due to their characteristics of low density, high oxidation resistance, and high operating temperature. However, TiAl alloys demonstrate brittleness at room temperature and possess low fracture toughness, which considerably restricts their practical applications [5,6,7,8]. Significant efforts have been devoted to the development of laminated metal composites (LMCs) that emulate the structure of nacre in shells, with the aim of enhancing the toughness and plasticity characteristics of TiAl alloy [9,10]. Shell nacre is a naturally occurring layered composite material characterized by multiple toughening mechanisms such as crack deflection [11], crack passivation [12], and interlayer pullout [13]. Similarly, LMCs consisting of alternating layers with varying levels of toughness and brittleness exhibit a synergistic combination of strength and toughness, leading to significant improvements in fracture toughness, fatigue performance, and impact resistance. The ductile layers within the composite play a crucial role in resisting the primary crack propagation and the development of the secondary cracks [14]. In contrast to monolithic TiAl or Ti alloys, metal–intermetallic laminate (MIL) composites offer an optimal combination of high strength, toughness, and stiffness [15,16]

Explosion welding [17,18], the rolling method [14,15,16], and hot pressing [15,19,20,21,22,23] are commonly used methods for fabricating MIL composites. Among these methods, vacuum hot pressing (VHP) is a novel one-step process that utilizes a controlled reaction at elevated temperatures and pressures. The physical and mechanical properties of MIL composites can be tailored via the selection of the appropriate matrix alloys and adjusting their thickness, thereby rendering VHP suitable for fabricating MIL composites with specific performance objectives [24]. Zhu [15] investigated the interface morphologies and mechanical properties of Ti-43Al-9V-Y/Ti6Al4V MIL composites prepared through VHP. The findings revealed that the fabricated composites exhibited superior tensile properties at room temperature compared to the monolithic TiAl alloy. The tensile strength of the composite is 654.48MPa, the elongation is 0.66%, and the fracture toughness is 31.37 MPam^1/2^, which indicate a significant enhancement compared to the TiAl alloy. Fan [14,21] fabricated Ti/Al laminate composites by using Ti and Al foils via VHP and determined the optimal layer thickness ratio by assessing both the interface bond and mechanical properties of the foils. Fan also researched the fatigue crack initiation, propagation paths, and fatigue crack growth rates of the Ti/Al laminate composites. Sun [25,26,27] fabricated Ti-TiAl MIL composites through VHP and identified that high densities of dislocations and substructures were primarily responsible for enhancing the strength of these composites. Tian [28] fabricated Ti/Al3Ti MIL composites with different ductile phase volume fractions through hot-pressing, revealing the relationship between the volume fraction of ductile phases, interfacial microstructural features, and macroscopic mechanical properties of laminated composites.

However, there are relatively few studies on the preparation of laminated materials using TiAl4822. In the present study, a Ti–48Al–2Cr–2Nb (TiAl4822)/Ti-6Al–4V (Ti6Al4V) MIL composite was manufactured through VHP. TiAl4822 was utilized as the strong layer. While Ti6Al4V, which exhibits commendable overall performance, served as the ductile layer, and was introduced to form MIL composites to improve the fracture toughness and room temperature ductility of the single TiAl4822. The microstructural evolution and interface bonding were thoroughly examined, followed by tensile and fracture toughness tests to assess the performance of the resulting MIL composite. The strengthening and toughening mechanisms of the composite were emphasized. The study in this paper can provide a new approach for the preparation of MIL composites. It can also further address the industrial application limitations of TiAl4822 alloy. Under the condition of meeting the requirements for part preparation, TiAl4822/Ti6Al4V MIL composites can be used to replace some TiAl4822 alloys, thereby enhancing the performance of the parts.

## 2. Experimental Procedures

### 2.1. Structural Design

**Figure 1:**
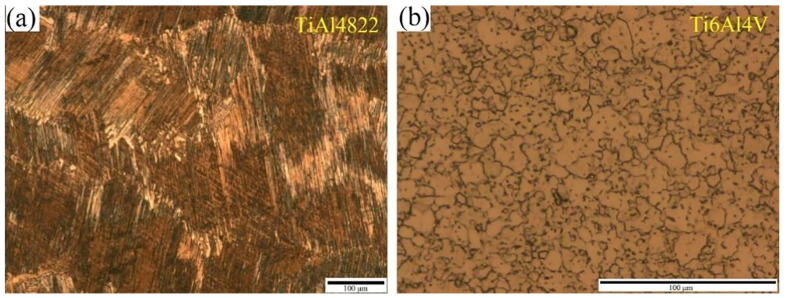
OM of the starting alloy: (**a**) TiAl4822; (**b**) Ti6Al4V.

**Figure 2:**
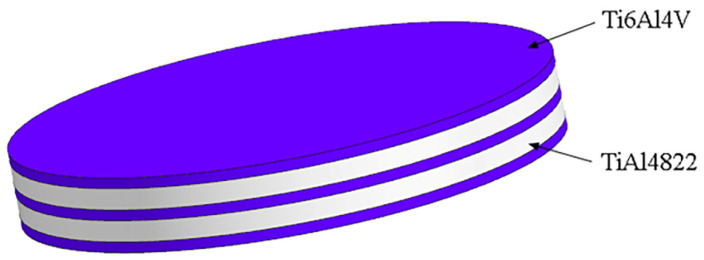
Schematic of the preform.

The TiAl4822 alloy with FL structure used in this study was prepared by plasma melting. Ti6Al4V foil (0.5 mm) was prepared by multi-pass rolling. The microstuctures of the initial alloys are shown in Figure 1. The colony size of TiAl4822 is approximately 200 µm, while the grain size of Ti6Al4V measures around 10 µm. Their chemical compositions are shown in Table 1. The TiAl4822 ingot was machined into several circular plates with a diameter of Φ50 mm and the thickness of 1 mm through wire electrical discharge. Similarly, the Ti6Al4V foil was processed into several circular plates of the same dimensions. The prepared specimens were pretreated in 100 vol.% HF solution for 2 min to eliminate oxide scales from their surfaces, followed by washing distilled water and drying. The thicknesses of the pretreated TiAl4822 circular plates were measured to be 0.9 mm. A laminate consisting of three layers of Ti6Al4V and two layers of TiAl4822 was fabricated in the sequence provided in Figure 2. The theoretical volume fraction of the Ti6Al4V layer was approximately 46% as calculated. When the volume fraction of Ti layer is 45%, the mechanical properties of MIL are optimized [29].

### 2.2. Sintering

**Figure 3:**
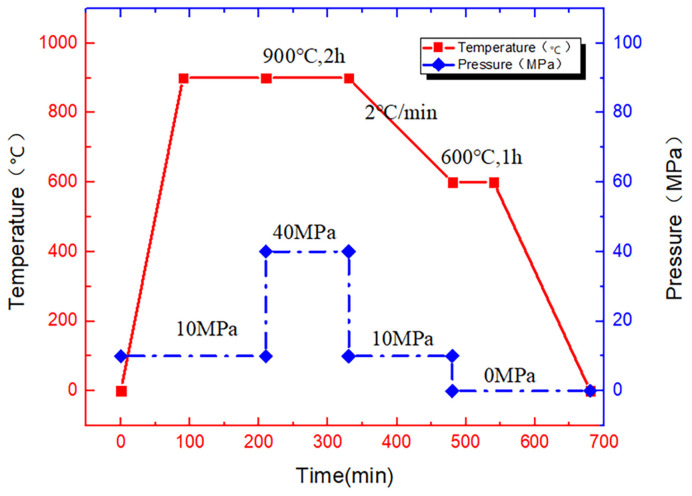
Schematic of VHP processing parameters.

The laminated preform was transferred to a VHP furnace (ZM-44-12Y) for the fabrication of the TiAl4822/Ti6Al4V MIL composite. The typical VHP configuration and processing parameters are illustrated in Figure 3. Wang [30] conducted a series of experiments using various combinations of temperatures, pressures, and holding times to determine the optimal parameters for preparing pore-free specimens by VHP. If the temperature or pressure is insufficient, or if the holding time or pressure-holding duration is too short, it may result in the formation of numerous pores within the composite material. Conversely, increasing the temperature, pressure, and holding time promotes grain growth in the alloy, widens the bonding interface region, and enhances both hardness and fracture strength. In this study, a set of relatively appropriate parameters was selected for the VHP experiment. The VHP process consists of three stages: the consolidation stage, the solid–solid reaction stage, and the annealing stage. During the consolidation stage, the temperature was elevated from room temperature to 900 °C at the heating rate of 10 °C/min and maintained for 120 min, while the pressure was maintained at 10 MPa. In the solid–solid reaction stage, the temperature was maintained at 900 °C for 120 min under the exerted pressure of 40 MPa. In this stage, the TiAl4822 and Ti6Al4V stacks were fully diffused and reacted. In the annealing stage, the pressure was released, and the temperature was reduced to 600 °C, held for 60 min, and then reduced to room temperature in the furnace. Annealing is beneficial for increasing the density of the composite, thereby improving mechanical properties [31]. Consequently, MIL composite consisting of three layers of Ti6Al4V and two layers of TiAl4822 are fabricated.

Specimens were cut from the fabricated TiAl4822/Ti6Al4V MIL composite using wire electrical discharge machining and subsequently embedded in epoxy resin. The specimens were initially ground using silicon carbide, then polished with 1.0 and 0.5 diamond suspensions, and subsequently etched in a solution comprising 5%HF + 15%HNO_3_ + 80%H_2_O (vol.%) for a duration of 8–12 s. Scanning electron microscopy (SEM, FEI Nova Nano SEM450, Hillsboro, OR, USA), energy dispersive X-ray spectroscopy (INCA 250X-Max 50, Oxford, UK), and X-ray diffraction (XRD, SMART APEX-Ⅱ, Billerica, MA, USA) were conducted for microstructural observation and phase identification. Transmission electron microscopy (TEM, Talos F200X, Hillsboro, OR, USA) and electron backscatter diffraction (EBSD, EDAX-TSL, Mahwah, NJ, USA) analyses were performed to characterize the phases and microstructures within interface regions.

### 2.3. Mechanical Property Measurements

**Figure 4:**
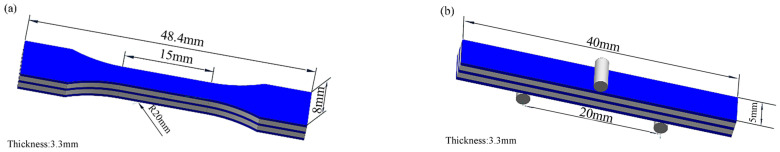
Schematic of the specimen: (**a**) tensile test; (**b**) three-point bending test.

Room temperature tensile tests provide a more accurate reflection of key performance indicators, including material strength and ductility. Three-point bending tests are employed to evaluate the material’s resistance to bending loads. Room temperature tensile and three-point bending tests were conducted on the TiAl4822/Ti6Al4V MIL composite by using an electronic universal testing machine (Zwick Z100, Ulm, BW, DE). The tensile loading rate was 0.1 mm/min, while the loading speed of the three-point bending test was set at 2 mm/min. The sample sizes are shown in Figure 4a,b.

**Figure 5:**
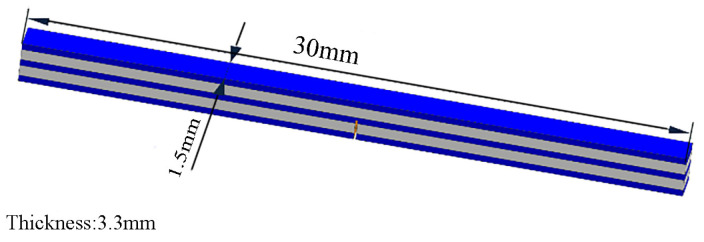
Fracture toughness testing specimen.

Fracture toughness reflects a material’s ability to resist the unstable propagation of cracks, and thus, to resist brittle fracture. The fracture toughness was tested on the CMT6103 electronic universal testing machine (Shenzhen, China). The dimensions of the test specimen are 1.5 mm × 3.3 mm × 30 mm, as shown in Figure 5. The cutting direction is aligned with the hot pressing direction with a cutting depth of 1.65 mm and a cutting width of 0.1 mm. The moving speed of the indenter is set at 0.5 mm/min.

The fracture toughness value was calculated using the following formula [15]:

(1) $$K_{IC}=\frac{3PL\sqrt{10a}}{200bh^{2}}\left[1.93-3.07\left(\frac{a}{h}\right)+14.53{\left(\frac{a}{h}\right)}^{2}-25.07{\left(\frac{a}{h}\right)}^{3}+25.08{\left(\frac{a}{h}\right)}^{4}\right]\left(MPa\cdot m^{\frac{1}{2}}\right),$$

where P is the maximum load value (N) when the specimen breaks, b is the specimen width (mm), L is the span (mm), a is the specimen notch depth (mm) and h is the specimen thickness (mm).

## 3. Results and Discussion

### 3.1. Microstructure Characterization

**Figure 6:**
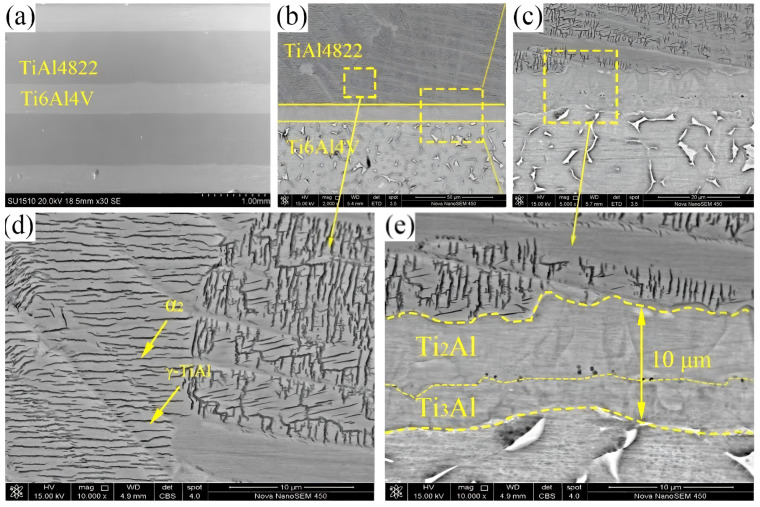
MIL micromorphology: (**a**) macro-organization interface and (**b**) MIL interface area image; (**c**,**e**) detailed drawing of the interface area; (**d**) microstructure of the TiAl4822.

The cross-sectional SEM images of the TiAl4822/Ti6Al4V MIL composite are illustrated in Figure 6a. Figure 6b illustrates the SEM image of the interface between the TiAl4822 and Ti6Al4V layers. The interface exhibits excellent bonding, with no visible cracks or voids. Fukutomi [32] fabricated Ti/TiAl laminated composites by using pure Ti and Al foil as raw materials and found that a significant number of Kirkendall pores were generated owing to the differing diffusion rates of titanium and aluminum atoms. The results in this work indicated that Kirkendall effects can be avoided by selecting TiAl4822 and Ti6Al4V as the raw materials in VHP processing. A similar result was also documented in the preparation of the Ti–43Al–9V–Y/Ti6Al4V MIL composite [15].

Figure 6c,e illustrate the formation of an interface layer with an approximate thickness of 10 µm between the TiAl4822 layer and the Ti6Al4V layer. The interface layer can be distinctly categorized into two regions: the region near TiAl4822 (with the thickness of approximately 6 µm) and the region near Ti6Al4V (with the thickness of approximately 4 µm).

**Figure 7:**
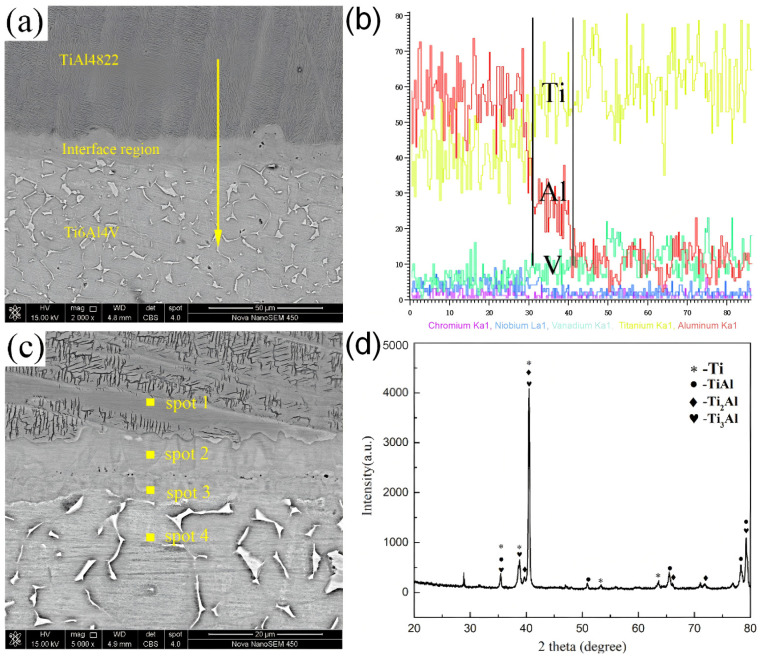
Phase composition of the TiAl4822/Ti6Al4V MIL composites: (**a**) scan line trace, (**b**) EDS line scan, (**c**) EDS spot scan, and (**d**) XRD spectrum.

The results of the XRD and EDS analysis of the TiAl4822/Ti6Al4V MIL composite are shown in Figure 7 and Table 2, respectively. The line scan energy spectrum analysis at the interface region is illustrated in Figure 7a,b. The formation of the interface can be attributed to the significantly higher aluminum content in the TiAl4822 layer compared to the Ti6Al4V layer, Al atoms diffuse from the TiAl4822 layer into the Ti6Al4V layer, leading to a gradual depletion of Al in the vicinity of the interface as shown in Figure 7b. Conversely, due to the higher titanium content in the Ti6Al4V layer relative to the TiAl4822 layer, Ti atoms diffuse from the Ti6Al4V layer into the TiAl4822 layer, resulting in Ti enrichment near the interface [30]. Ultimately, the interface forms through metallurgical bonding rather than simple mechanical bonding, thereby ensuring robust interfacial strength. Eventually, as shown in Figure 7c, an interface region formed between the TiAl4822 and Ti6Al4V layers through metallurgical bonding [15]. Consequently, the bonding strength of the interface can be guaranteed.

The XRD patterns of the interface region are illustrated in Figure 7d. The results of XRD analysis revealed the presence of the α-Ti, β-Ti, $\alpha_{2}$-Ti_3_Al, Ti_2_Al, and γ-TiAl phases in the interface. The typical phases of the TiAl4822 alloy consisted of $\alpha_{2}$ and γ phases as marked in Figure 6d. Meanwhile, the typical phases of Ti6Al4V were the α and β phases [33]. The line scan result in Figure 7b indicated that the aluminum element content within the interface region varied between 20% and 36%. Based on the Ti–Al binary phase diagram [34], it can be inferred that the $\alpha_{2}$ phase existed in the interface area. This result, when considered alongside the EDS analysis results in Table 2, indicated that the two regions in the interface layer consist of the $\alpha_{2}$-Ti_3_Al and Ti_2_Al phase, respectively.

**Figure 8:**
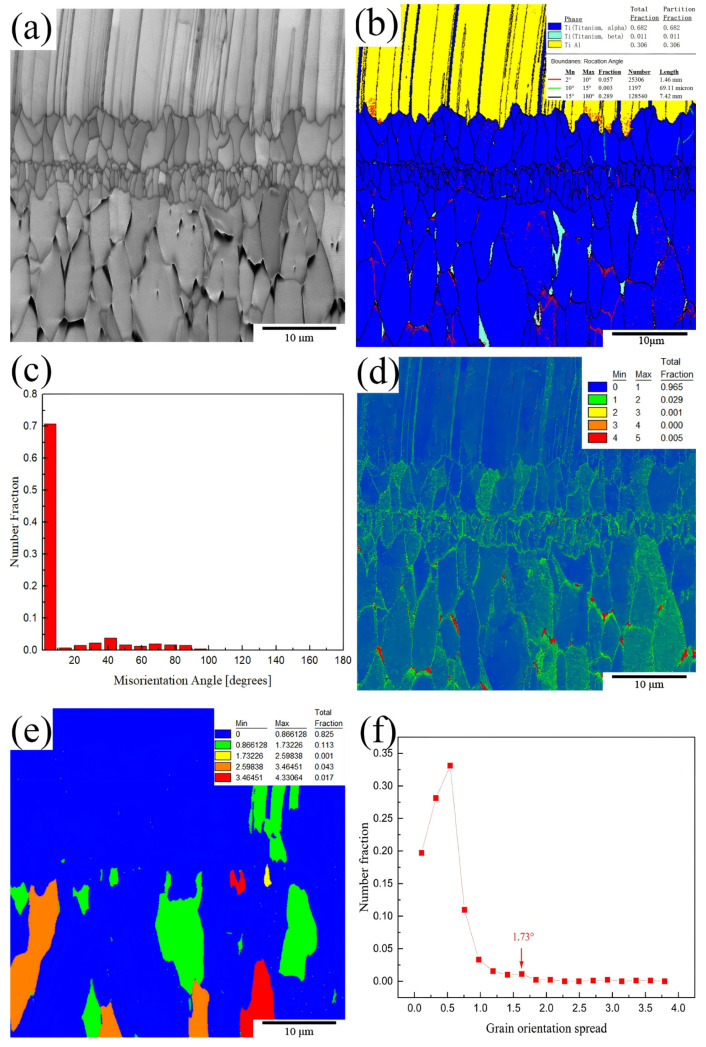
EBSD analysis of the TiAl4822/Ti6Al4V MIL cross-section: (**a**) grain morphology, (**b**) phase distribution and grain boundary diagram, (**c**) grain angular orientation difference, (**d**) KAM map of MIL, (**e**) image quality mapping of GOS values and their volume fractions, (**f**) critical GOS value.

Figure 8 shows the EBSD results for the interface region of the TiAl4822/Ti6Al4V MIL composite. Figure 8a,b indicate that the phases within the TiAl4822 alloy matrix contained coarse γ laths (yellow) and fine lath-like $\alpha_{2}$, which are uniformly distributed among the γ laths. The phases in the Ti6Al4V alloy matrix are the equiaxed α phase (blue) and the β phase (light green) at the α phase grain boundaries. As previously discussed, the phases in the interface region are $\alpha_{2}$-Ti_3_Al and Ti_2_Al. The statistical results of the phase distribution (Figure 8b) and the grain orientation difference (Figure 8c) reveal that the proportions of $\alpha /\alpha_{2}$ phase, γ-TiAl, and β-Ti are 68.2%, 30.6%, and 1.1%, respectively. Among these phases, high-angle grain boundaries account for 28.9%, whereas small-angle grain boundaries account for 70.8%. Notably, the 2–15° grain boundaries within the small-angle grain boundaries only account for 0.57%, whereas the 1–2° subgrain boundaries account for 65.1%. The easy occurrence of dislocation plugs at grain boundaries hindered plastic deformation, while the abundance of subgrain boundaries facilitates the movement of dislocations.

The DRX temperatures of Ti6Al4V and TiAl4822 can be attained during hot pressing, resulting in the formation of equiaxed and undistorted DRX grains within the bonding regions. The unsmooth surface on the joint surface under hot pressing pressure [35] facilitated the development of DRX grains. A significant accumulation of dislocations accumulated on the surfaces of the joint. The generation of deformation energy serves as the driving force for recrystallization. Fine grain layers formed close to Ti6Al4V matrix, while the grain size was rather coarse in the layers close to TiAl4822 matrix. The nucleation and growth of the recrystallized grains within the interface layer as a function of increased holding time facilitated interface migration and formed the interface layer, as illustrated in Figure 8a. Figure 8e provides the grain orientation spread (GOS) value of the degree of lattice distortion. The blue and green regions in Figure 8d present the low density of dislocations associated with dynamic recrystallization (DRX) and static recovery. The rather low kernel average dislocation (KAM) observed after hot pressing suggested that the stored strain energy in these regions was dissipated through recrystallization and static recovery during the hot pressing process. Thus, low dislocation density can be resulted.

**Figure 9:**
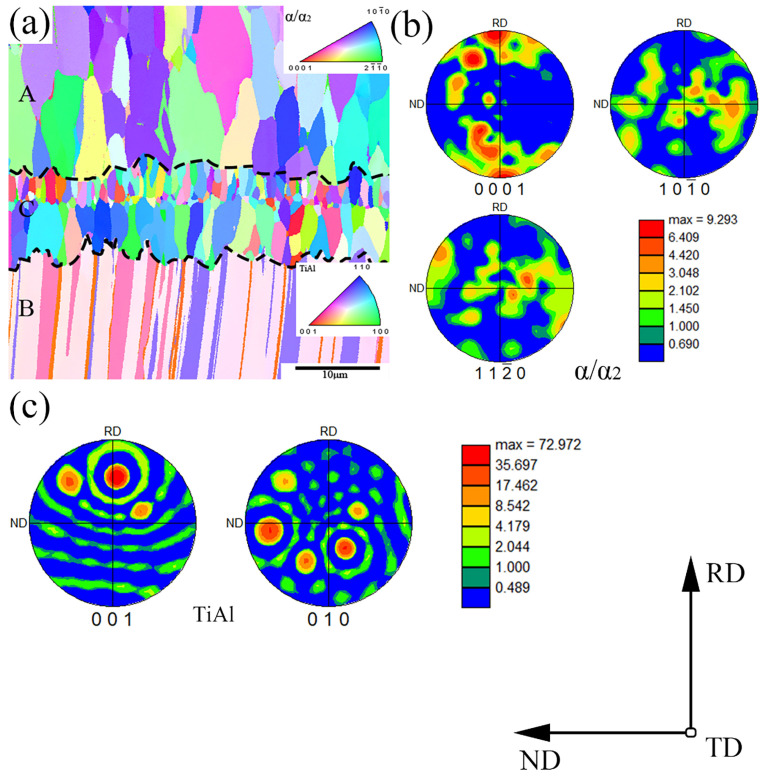
Texture distribution of TiAl4822/Ti6Al4V MIL composite: (**a**) EBSD inverse pole figure of region A: Ti6Al4V martrix, regionn B: TiAl4822 martrix and region C: interface layer, (**b**) EBSD pole figure of α/α2, (**c**) EBSD pole figure of TiAl.

Figure 9 shows the interface layer of the TiAl4822/Ti6Al4V layered composite, along with the texture distribution of the matrix grain adjacent to the interface layer. The RD direction is aligned with the hot pressing direction, while the TD direction is orthogonal to the interface layer direction. Additionally, the ND direction is perpendicular to the plane formed by RD and TD. As shown in region A (Figure 9a) and Figure 9b, the equiaxed α/α2 grain texture of the Ti6Al4V matrix after hot pressing exhibits a weak characteristic. In contrast, TiAl4822 exhibits a strong texture due to its lamellar structure, as shown in region B and Figure 9c. The grain orientation equiaxed Ti_3_Al and Ti_2_Al grain texture at the interface layer exhibits a weak texture, and the grain orientation is anisotropic, as shown in region C and Figure 9b. The local texture analysis indicates that the TiAl sheet (pink) within region B demonstrates a strong {001}-preference orientation along the RD direction. In contrast, the grain texture of the Ti6Al4V matrix and interfacial layer region is weak and the orientation is random.

**Figure 10:**
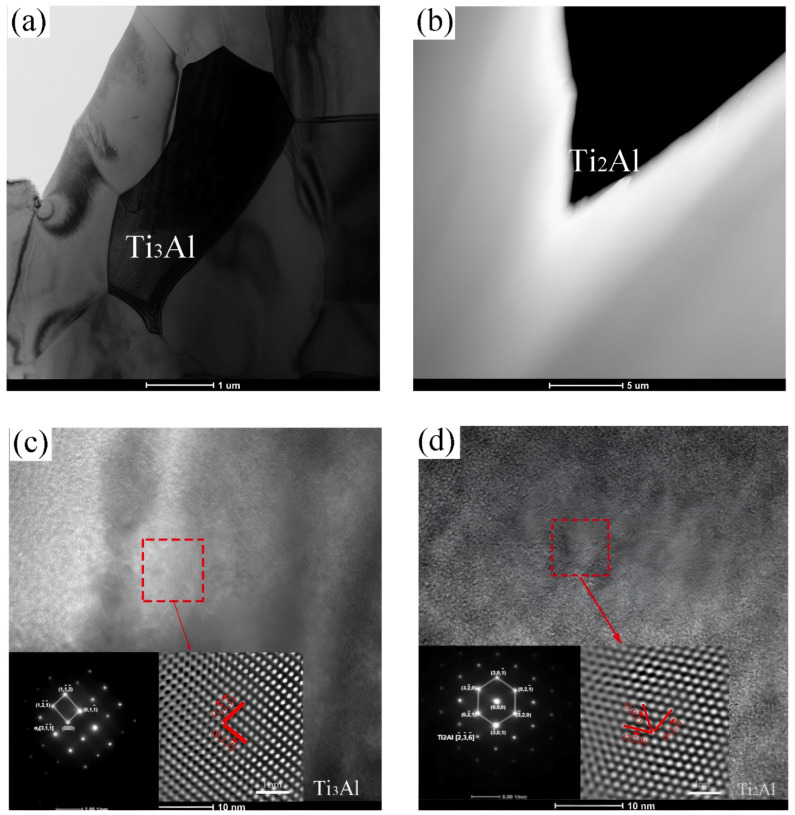
Microstructural TEM images of the TiAl4822/Ti6Al4V MIL: (**a**) Ti_3_Al phase, (**b**) Ti_2_Al phase, (**c**) high-resolution image and diffraction spots of Ti_3_Al (**d**) high-resolution image and diffraction spots of Ti_2_Al.

XRD diffraction analysis revealed that the presence of α_2_-Ti_3_Al and Ti_2_Al phases within the microstructure of the composite was known. To further validate these phase species, a detailed examination of the composite interface layer was conducted using TEM. Figure 10 presents both the TEM image of the composite interface layer and its corresponding SAED image. Figure 10a,b are the $\alpha_{2}$-Ti_3_Al phase near the Ti6Al4V layer and the hexagonal Ti_2_Al phase near the TiAl layer, respectively. Figure 10c,d are the high-resolution and diffraction spot images of the Ti_3_Al and Ti_2_Al phases, respectively.

### 3.2. Mechanical Properties

**Figure 11:**
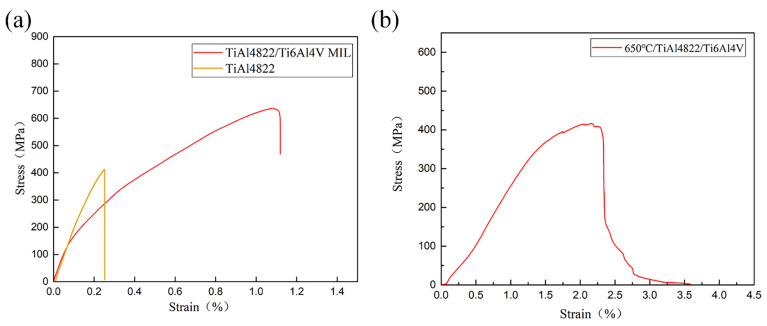
Tensile stress–strain curves of: (**a**) TiAl4822/Ti6Al4V MIL composite and TiAl4822 at room temperature; (**b**) TiAl4822/Ti6Al4V MIL composite at 650 °C.

Figure 11 illustrates the tensile stress–strain curves of the TiAl4822/Ti6Al4VMIL composite at both room temperature and 650 °C. The tensile stress–strain curve was also obtained for comparison. The composite exhibits a tensile strength of 636.9 MPa and an elongation of 1.13%. The tensile strength is 416.6 MPa at 650 °C, and the tensile strain is 2.30%. The initial TiAl4822 alloy exhibits a room temperature tensile strength of 411.8 MPa and an elongation of 0.25%. Notably, compared to the initial TiAl4822, there is a significant enhancement in the elongation of tensile strength at room temperature for the composite, indicating that the stacked structure effectively mitigates brittleness in TiAl alloys. In comparison to the work of Zhu [15], this paper exhibits a slight decrease in tensile strength; however, both the elongation and fracture toughness exhibit significant improvements. The specific values are detailed in Table 3. It can be observed that some fluctuations present at the end of the room temperature stress–strain curve. The cracks initiated and propagated continuously within the brittle TiAl4822 layer, while the bearing limit was approached. When the maximum value was reached, failure occurred in the TiAl layer, resulting in a decrease in tensile strength, which corresponds to a slight decrease in the tensile stress–strain curve. Subsequently, the ductile Ti6Al4V layer continued to bear the load, leading to a slight increase in displacement. Ultimately, as the load increased in intensity, the Ti6Al4V layer gradually succumbed to failure until the composite was completely fractured.

**Figure 12:**
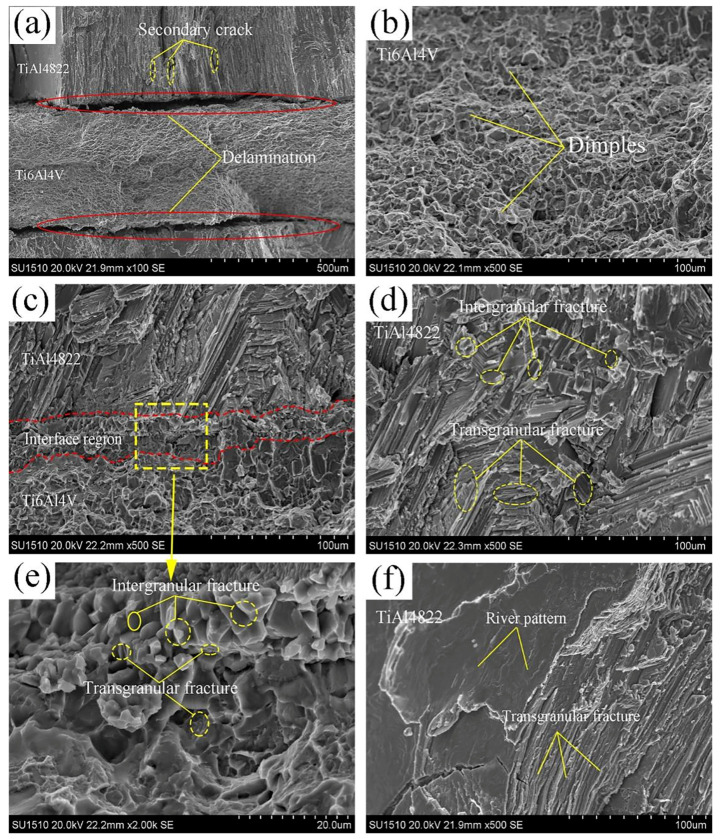
Fractographies of the TiAl4822/Ti6Al4VMIL composite: (**a**) fracture surface of the cross-section, (**b**) morphology of the Ti6Al4V ductile fracture, (**c**,**e**) fracture morphology of the interface region, and (**d**,**f**) brittle cleavage and quasi cleavage fracture morphology of TiAl4822.

Figure 12 shows the tensile fracture morphology of the TiAl4822/Ti6Al4VMIL composite. As shown in Figure 12a, the different deformability of TiAl4822 and Ti6Al4V alloys resulted in delamination at the interface area during the tensile process. Additionally, secondary cracks were observed within the TiAl matrix near the interface area. Figure 12b shows the fracture morphology of the Ti6Al4V layer. There are numerous dimples and ductile intergranular fractures in the fracture section, indicating that ductile fracture was the main fracture mold of Ti6Al4V layer. As shown in Figure 12d, within the TiAl4822 matrix far away from the interface layer, two distinct fracture modes are observed: intergranular fracture and transgranular fracture. In Figure 12f, the fracture surface of TiAl4822 matrix exhibits river patterns, which are characteristic of typical brittle cleavage fractures. It can be observed from Figure 12c,e that the fracture mode observed in the interface region predominantly exhibits brittle cleavage fracture, which encompasses both intergranular and transgranular fractures.

In Figure 8a, it can also be observed that there are fine grains (1 μm to 5 μm) in the interface layer. Fine grains, coupled with a significant grain boundary area, effectively inhibit intergranular slip. Consequently, the interface can effectively absorb fracture energy, thereby enhancing both the overall strength and plasticity of MIL composites. Furthermore, the interaction among the ductile-brittle structure, secondary cracks, delamination, and the fine grain structure within the interface region promotes crack passivation, crack deflection, and load transformation. As a result, the tensile strength and fracture toughness of MIL composites are significantly improved.

**Figure 13:**
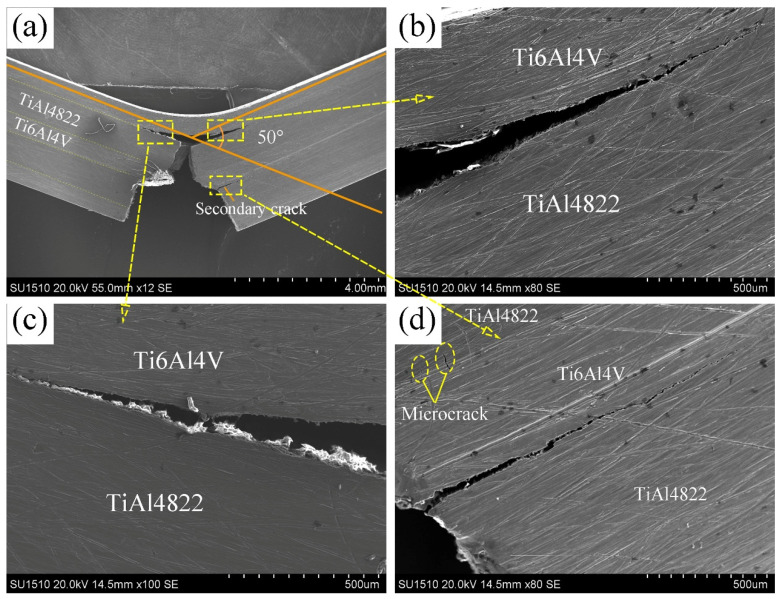
Crack propagation characteristics of TiAl4822/Ti6Al4V MIL fracture toughness test specimen in the direction parallel to the hot pressing direction: (**a**) overall specimen morphology; (**b**–**d**) Local crack growth path.

According to the calculations based on Formula (1), the fracture toughness value of TiAl4822/Ti6Al4V MIL composites, measured in the direction parallel to the hot pressing, is 33.15 MPam^1/2^. Due to the exceptional toughness of Ti6Al4V, as well as the existence of interface regions, the MIL exhibits superior fracture toughness compared to the as-cast TiAl4822 alloy (7.2 MPam^1/2^). Figure 13 shows the crack propagation characteristics of the TiAl4822/Ti6Al4V MIL fracture toughness specimen. As shown in Figure 13a, the unilateral notch of the fracture toughness sample is positioned on the Ti6Al4V layer. Under the application of external loads, the primary crack initially develops at the single-sided notch. As the load increases and the primary crack propagates upward toward the interface region, secondary cracks preferentially initiate at the interface between the TiAl4822 layer and the Ti6Al4V layer. These secondary cracks subsequently propagate laterally along this interface rather than parallel to the direction of the applied load. This crack propagation behavior is consistent with the findings reported by Zhu [15]. Due to the high brittleness of TiAl4822, the crack resistance is rather small. Therefore, the TiAl4822 layer presented a brittle fracture when the main crack propagated. No crack deflection was observed during this process. Additionally, exist some small cracks that were identified at the interface area between this layer of TiAl4822 and the middle layer of Ti6Al4V, as shown in Figure 13d. The crack continued to propagate until it was blocked by the top layer of Ti6Al4V, attributed to its superior room temperature toughness. Ultimately, the main crack is fractured and the secondary crack started to expand further along the interface layer to both sides, as shown in Figure 13b,c. From Figure 13a, it is observed that the Ti6Al4V layer of the top layer exhibits considerable deformation with the bending angle of approximately 50°. During crack propagation, crack deflection and the generation of micro cracks can dissipate the energy required for crack propagation, thereafter, improving the fracture toughness of MIL.

**Figure 14:**
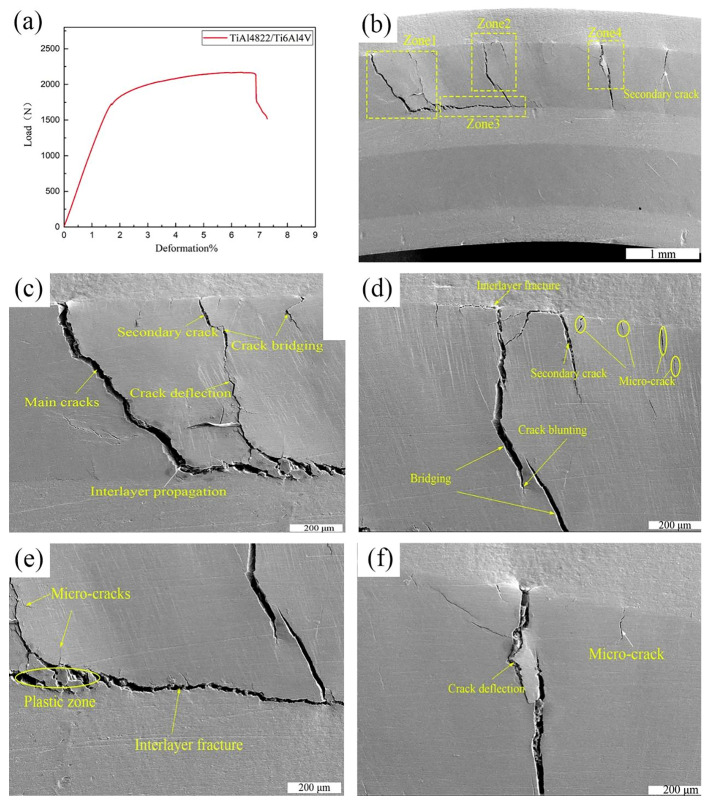
Load deformation curve and crack propagation characteristics of the TiAl4822/Ti6Al4V MIL composite: (**a**) load deformation curve, (**b**) crack growth morphology, (**c**–**f**) enlarged view of the local area in Figure 12b.

A three-point bending test was conducted on the TiAl4822/Ti6Al4V MIL composite in the direction parallel to hot pressing direction. The bending strength was measured as 1114.1 MPa, while the deformation reached 7.28%. Figure 14 shows the load–deformation curve and crack propagation characteristics of the composites. TheTiAl4822/Ti6Al4V MIL composite did not experience complete fracture during the testing process. Given that the outer layer bore the maximum bending stress, cracks predominantly initiated in the outermost layer of TiAl4822 matrix, as presented in Figure 14b. The Ti6Al4V layers did not fracture due to their excellent ductility. Initially, minute cracks initiated at the upper surface of the outer TiAl4822 layer and propagated downward. With the increasing loading, these cracks propagated within the TiAl4822 layer and formed the main cracks throughout the whole layer (Figure 14c). Subsequently, the presence of the Ti6Al4V layer impeded the propagation of these cracks, resulting in their deflection and progression along the interface region. It also can be observed that secondary cracks initiated within the TiAl4822 layer. Concurrently, microcracks initiated in the loading direction due to the intrinsic brittleness of the TiAl4822 alloy (Figure 14d). Energy absorption phenomena, such as crack blunting, bridging, and deflection can be observed in Figure 14c,d. Additionally, local plastic deformation and interface delamination were also observed as shown in Figure 14e,f. Energy dissipation will increase with the increase in crack propagation paths, thereby the fracture toughness of the MIL composite will consequently be enhanced.

## 4. Conclusions

TiAl4822/Ti6Al4V MIL composites were prepared through VHP. The following conclusions can be obtained:

The interface region of the fabricated TiAl4822/Ti6Al4V MIL composite exhibits excellent bonding without evident defects. Atomic diffusion between the two alloys results in a dense interfacial layer approximately 10 μm thick, with grains in this region exhibiting no distinct texture. KAM and GOS analyses reveal a relatively low dislocation density within the MIL composite. Dislocations present in these regions are effectively consumed during dynamic recrystallization (DRX) and subsequent recovery processes. A significant number of subgrain boundaries emerge during recovery, which facilitate dislocation movement and enhance the plasticity of the MIL composite. Additionally, the grains in the interface region and the brittle–tough nature of Ti6Al4V and TiAl4822 intermetallic compounds promotes mechanisms such as crack passivation, crack deflection, and load transfer, thereby improving the overall strength and ductility of the MIL composite. The tensile strength of the MIL composite is measured at 636.9 MPa, with a tensile strain of 1.13%, representing a substantial improvement over the TiAl4822 alloy. Furthermore, the room temperature bending strength of TiAl4822/Ti6Al4V MIL was measured at 1114.1 MPa. The main crack propagation first occurred within the TiAl4822 layer, subsequently deflecting towards the interface layer due to the resistance offered by Ti6Al4V. Toughening mechanisms, including secondary cracks, microcracks, and crack bridging in both the TiAl4822 layer and the interface layer, contributed to the energy dissipation of the MIL composite.

## Figures and Tables

**Table 1:** Chemical composition of the original foil.

Materials	Composition (%)
TiAl4822	Ti: Margin Al 48.1, Cr 1.92, Nb 1.95, Fe < 0.03, C < 0.03, O 0.24, N < 0.03, H 0.04
Ti6Al4V	Ti: Margin Al 5.5–6.8, V 3.5–4.5, Fe < 0.3, C < 0.1, N < 0.05, H < 0.015, O < 0.2

**Table 2:** EDS analysis of the points in Figure 7c (at.%).

Point No.	Ti	Al	Nb	Cr	V	Phase
Spot1	49.43	46.17	2.26	2.14		γ-TiAl
Spot2	67.40	28.69	1.92		1.98	Ti_2_Al
Spot3	76.39	22.02			1.59	$\alpha_{2}$-Ti_3_Al
Spot4	85.87	10.91			3.22	Ti

**Table 3:** Tensile strength and strain of the TiAl4822/Ti6Al4V MIL composite.

Specimen	Tensile Strength (MPa)	Tensile Strain (%)
TiAl4822/Ti6Al4V MIL (RT)	636.9	1.13
TiAl4822 (RT)	411.8	0.25
Ti43Al9V-Y/Ti6Al4V LMC (RT) [11]	654.3	0.66
TiAl4822/Ti6Al4V MIL (650 °C)	416.6	2.30

## Data Availability

The original contributions presented in this study are included in the article. Further inquiries can be directed to the corresponding author.

## Abbreviations

The following abbreviations are used in this manuscript:

MIL	Metal–intermetallic laminate
VHP	Directory of open access journals
DRX	Three letter acronym
LMCs	Linear dichroism
GOS	grain orientation spread
KAM	Kernel average dislocation
SEM	Scanning electron microscopy
TEM	Transmission electron microscopy
XRD	X-ray diffraction
EBSD	Electron backscatter diffraction
